# Strategies to improve uptake and adherence of non-pharmacologic interventions for orthostatic hypotension in older people: a qualitative study

**DOI:** 10.1007/s41999-022-00632-6

**Published:** 2022-03-14

**Authors:** Lisa Robinson, Ruth Pearce, James Frith

**Affiliations:** 1 The Newcastle upon Tyne Hospitals NHS Foundation Trust, Newcastle upon Tyne, UK; 2 NIHR Newcastle Biomedical Research Centre and Population Health Sciences Institute, Newcastle University, Newcastle upon Tyne, UK

**Keywords:** Orthostatic hypotension, Postural hypotension, Treatment adherence and compliance, Conservative treatment, Behaviour and behaviour mechanisms

## Abstract

**Purpose:**

Non-pharmacologic therapies are a safe and effective treatment for orthostatic hypotension (OH) in older adults. However, adherence to non-drug therapies is challenging and may require specific behaviour change approaches to promote uptake and adherence. The study aim is to identify specific behavioural change techniques to promote uptake and adherence with non-pharmacologic interventions for older adults with OH.

**Methods:**

Forty semi-structured, qualitative interviews were performed in 25 older adults with OH. Each participant experienced bolus-water drinking, physical counter-manoeuvres and compression garments during two efficacy studies. Emergent themes were identified through framework analysis, based on The Behaviour Change Technique Taxonomy.

**Results:**

Several themes to encourage uptake and adherence arose. Motivation to adhere with an intervention may be improved by demonstrating its effectiveness, either through symptom monitoring or biofeedback. Practising or rehearsing how to use an intervention may improve self-efficacy and promote habit formation. Embedding therapies into daily life so that they become second nature was felt to be a useful strategy. Educating older adults about why they are being asked to use a therapy and demonstrating how to use it is important. More specific barriers may be overcome by encouraging a personal problem-solving approach.

**Conclusion:**

These specific behaviour change techniques, derived by older people with OH and based on evidence-based approaches, provide useful strategies to improve the uptake and adherence of non-drug therapies in the treatment of OH.

**Trial registration:**

ISRCTN15084870.

## Key Summary Points

**Aim:**

To identify specific behavioural change techniques to promote uptake and adherence with non-pharmacologic interventions for older adults with OH.

**Findings:**

Specific behaviour change strategies, derived from older people with orthostatic hypotension, include biofeedback, rehearsal, embedding into daily routine and patient education.

**Message:**

Evidence-based behaviour change strategies may be used to improve uptake and adherence to non-drug therapies for older people with orthostatic hypotension.

## Introduction

### Background

Orthostatic hypotension (OH) is a disabling condition characterised by a significant drop in blood pressure on standing upright. It is particularly relevant to older people in which it is very common, because it can lead to disabling symptoms, falls, reduced quality of life and an increased risk of cardiovascular disease and death [1, 2]. Older adults with OH prefer non-pharmacologic management, in the hope of avoiding increased medication burden [3]. Recent studies have demonstrated that non-pharmacologic therapies are safe and efficacious, including bolus-water drinking, physical counter-manoeuvres and compression garments [4].

It is naive to focus solely on the efficacy of non-pharmacologic treatment strategies without considering measures to ensure adequate uptake and adherence. Such interventions may be considered complex because multiple components/behaviours are involved in their adherence. Changing behaviour requires an understanding of the influences on behaviour and the context in which they occur. These factors are typically complex, involving many interacting components, making them challenging to implement in practice [5].

Attempts to identify the active components associated with uptake and adherence with non-pharmacologic interventions for older adults with OH are necessary to better understand the effects and mechanisms of behaviour change interventions and to inform the development of more effective implementation strategies. A method rapidly gaining popularity for this purpose is the reliable characterisation of specific behaviour change techniques. Behaviour change techniques are the observable, replicable, and irreducible components of an intervention designed to alter or redirect causal processes that regulate behaviour; that is, a technique is proposed to be an ‘active ingredient’ (e.g. feedback, self-monitoring, and reinforcement) [6].

### Objective

To identify specific behavioural change techniques to promote uptake and adherence with non-pharmacologic interventions for older adults with OH.

### Conceptual framework

Behavioural change is more effective if interventions are based on principles drawn from evidence and theories of behaviour and behaviour change [7]. The Behavioural Change Technique Taxonomy (BCTT v1) is an extensive, consensually agreed hierarchically-structured taxonomy of 93 techniques clustered into 16 groups, providing a robust framework for reliable and systematic specification of behaviour change interventions [8].

## Methods

### Study design

A qualitative analysis was conducted using data from a two-stage phase 2 efficacy study evaluating non-pharmacologic therapies for OH in older people [4, 12]. The first stage of the study evaluated the efficacy, safety and tolerability of different therapies (bolus water drinking, compression stockings, abdominal compression, physical counter-manoeuvres) used in isolation. The second stage evaluated therapies in combination.

**Fig. 1:**
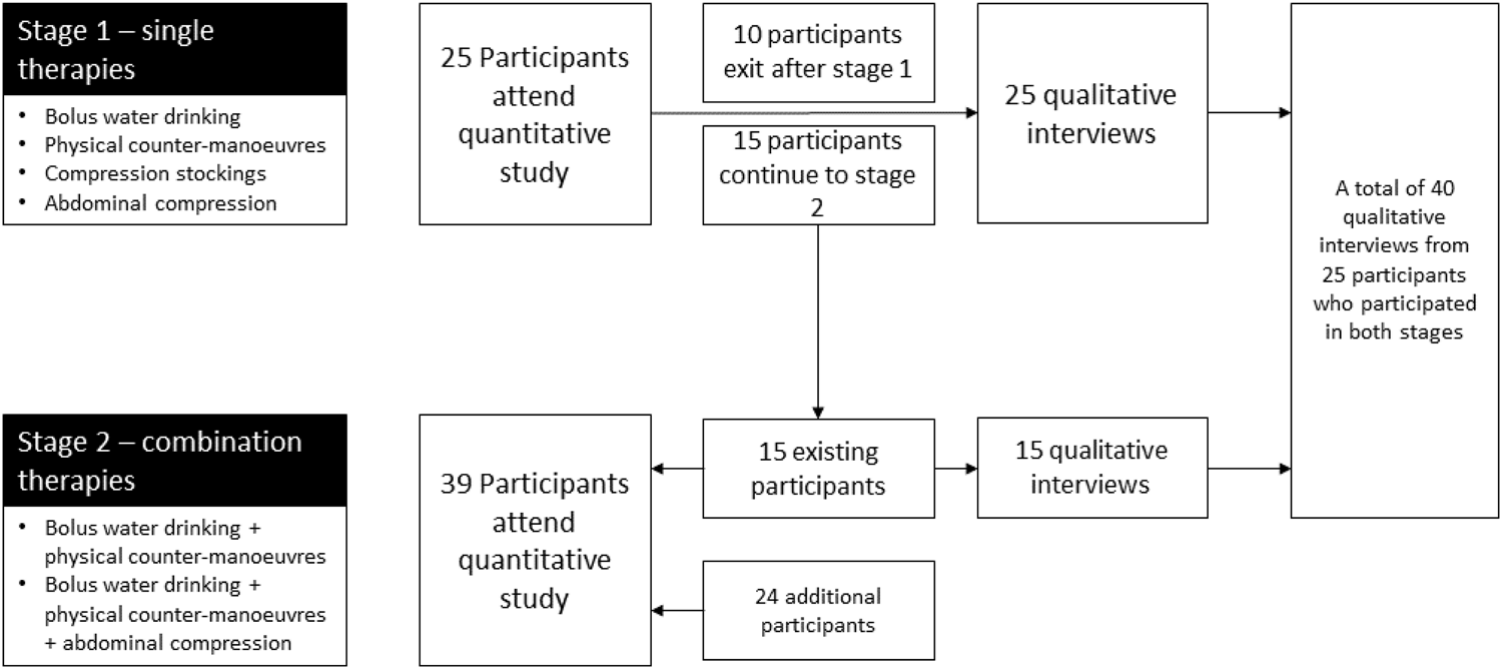
A summary of recruitment to qualitative interviews

Semi-structured interviews were conducted with all participants (*n* 25) who participated in the first stage of the efficacy study and were repeated in those who returned to experience combination therapies (*n* 15). Interviews were conducted between February and December 2016. Figure 1 summarises the recruitment to the two stages of the study.

### Setting and participants

All participants were aged 60 years or over and had a clinical diagnosis of OH in keeping with international diagnostic criteria [13]. Potential participants were excluded if they had contraindications to any of the therapies under evaluation. Participants were identified in a UK secondary care clinic in Northeast England.

Twenty-five individuals participated in stage 1 (therapies used in isolation) of the efficacy study; this number was anticipated to be large enough to capture a wide variety of data for both quantitative and qualitative analysis. As the second stage (therapies delivered in combination) required an increase in sample size for the quantitative analysis, an additional 24 participants were recruited from the outpatient hospital clinic to receive the non-pharmacological therapies in combination. However, only participants taking part in both stages of the study were asked to participate in a further interview, to ensure participants had experience of using all of the interventions. The final sample for the qualitative component therefore consisted of 40 interviews (25 participants who experienced single therapies, with 15 of these contributing to an additional interview focussing on therapies in combination).

### Intervention

Participants experienced each of the following therapies, to which researchers and participants were unblinded. For information, the response rate to each therapy has been included below (defined as reducing the postural systolic BP drop by at least 10 mmHg)as follows:

1. Stage one
2. Bolus water drinking: 480 ml of room-temperature tap water, to consume as much as possible within 5 min. Response rate 56% (95% confidence interval [CI] 37–74).
3. Physical counter manoeuvres: upon standing upright, participants were encouraged to tense their lower limb and abdominal muscles [14]. Response rate 44% (95% CI 26–63).
4. Compression stockings: full leg length, grade 2 compression stockings (23–32 mmHg, mediven plus). Response rate 32% (95% CI 16–51).
5. Abdominal compression: an elasticated belt (promedics pro-tem belt), applied (at 10 mmHg) to the participant’s abdomen and pelvis). Response rate 52% (95%CI 33–71).

2. Stage two

- Bolus water drinking + physical counter-manoeuvres. Response rate 38% (95% CI 34–63).
- Bolus water drinking, physical counter-manoeuvres + abdominal compression. Response rate 46% (95% CI 31–62).

### Quantitative data collection and analysis

Participants attended the hospital out-patient clinic to assess cardiovascular responses to interventions. For the purposes of providing descriptive data on the cohort’s level of co-morbidity, the Charlson Cormorbidity Score was reported alongside the number of medications [15]. Symptom severity was captured using the Orthostatic Hypotension Questionnaire [16]. Supine and standing BP were recorded using non-invasive methods (Taskforce, CNSystems). These methods are described in more detail elsewhere [4, 12].

### Qualitative data collection

There were three aims as follows: to identify the acceptability of and the barriers to the non-drug interventions and to explore potential solutions to these barriers. The acceptability of non-drug therapies are described in detail elsewhere [17].

To aid recall, the interviews were conducted as soon as possible after the quantitative component (median 8 days, interquartile range 7–14 days). Participants were offered the opportunity to be interviewed in their own homes in the hope that participants would speak more openly about their experiences. Twenty interviews from stage 1 were conducted in the participants’ own home, with the remaining five participants choosing to return to the hospital clinic. Of the 15 participants from Stage 2, all chose to be interviewed in clinic immediately after experiencing the combination therapies because the time burden was much less. Figure 1 summarises the recruitment to the two stages of the study.

The semi-structured interviews were conducted, audio-recorded, transcribed verbatim and anonymised by an experienced qualitative researcher [RP]. The interview schedule consisted of a single open-ended question about each intervention, ‘*how did you find [intervention] as a potential treatment?’* with follow-up questions to be used as prompts if needed. Participants were asked for suggestions that could help older adults with OH to adhere with the non-pharmacologic interventions. The average length of the interviews was 20 min (range 11–43 min).

## Qualitative data analysis

Interview transcripts were analysed using a modified framework analysis, specifically focussing on potential strategies to improve uptake and adherence [18]. Framework analysis allows both emergent data themes and the explicit recognition and use of a priori issues in the analytical framework. Framework analysis is increasingly being used in health services research, and it fitted the aims of this analysis due to the predefined areas under investigation, while remaining open to the emergence of further topics and themes. The BCTT v1 provided the initial coding framework or ‘indexing structure’ [18].

The specific steps undertaken during this analysis were as follows:

***Familiarisation: ***The researchers (LR, RP, JF) developed an initial sense of the data by reading through a sample of the transcripts.

***Identifying an initial coding framework: ***Two researchers (LR, RP) independently recorded their impressions and deductive themes. The researchers discussed these initial impressions in relation to their knowledge of the BCCT v1 and agreed on an initial coding framework.

***Indexing: ***The transcripts were sifted by RP, quotes were highlighted, and comparisons were made both within and between the interview data. During this stage, the data were labelled using the BCCT v1 groupings and behavioural change techniques for subsequent retrieval and exploration.

***Charting: ***Selected quotes were lifted from their original context and rearranged under the emerging coding framework. This process proved a valuable stage in helping to manage the data, making sense of what was going on by getting rid of extra and irrelevant data. Whilst charting, RP made note of any changes to the analytical framework. The evolving coding framework was discussed with the research team [LR, RP, JF] at regular data analysis meetings. Illustrative quotes were tagged and managed using Microsoft Word.

***Mapping and Interpretation: ***We considered this data alongside the interventions which were evaluated in order to generate clinically relevant conclusions and recommendations.

### Permissions

All participants confirmed informed consent in writing. The study was reviewed by Newcastle and North Tyneside 2 Research Ethics Committee who granted ethical permissions (15/NE/0308).

## Results

The demographic details of the cohort are summarised in Table 1. Seven specific behavioural change techniques were identified, which are found within four main themes of the BCT. These are summarised in Table 2 and described in more detail below.

**Table 1:** Participant demographic data

Demographic	
Age (years)^a^	74 (60–92)
Female/male	10/15
Charlson comorbidity score*	4 (3–8)
Hypertension	5
Type 2 diabetes	4
Parkinson’s disease	2
Multisystem atrophy	1
Number of regular medications	4 (0–13)
Fludrocortisone (N)	5
Midodrine (N)	3
Orthostatic blood pressure drop^b^	
Systolic	41 (22)
Diastolic	19 (13)
Orthostatic symptom severity^b^	
Total symptom score	6 (0–51)
Dizziness	2 (0–9)

^a^The median is displayed with the range for non-parametric data; ^b^the mean is presented with the standard deviation for normally distributed data

**Table 2:** Summary of emergent themes

Theme 1—Shaping knowledge
1.1 Instruction on how to perform the behaviour
1.2 Behavioural experiments
Theme 2—Feedback and monitoring
2.1 Self-monitoring of outcome of behaviour
2.2 Biofeedback
Theme 3—Repetition and substitution
3.1 Behavioural practice and regulation
3.2 Habit formation
Theme 4—Goals and planning
4.1 Problem solving

### Theme 1—Shaping knowledge

#### 1.1 Instruction on how to perform the behaviour

The BCT describes this technique as advising or agreeing on how to perform the behaviour e.g. the patient attends an educational event such as a group class.

This technique was a particularly common theme arising from the data which suggested that if older adults with OH understood why they were being asked to use the therapies they would be more likely to try them and adhere to them.

*I would tell them [people with OH] the advantage of wearing them [compression garments]*. (001)

*I could wear them [compression garments] if I thought there was an advantage with them*. (022)

*I knew I was drinking the water for a purpose... knowing that it was doing me some good*. (003)

In addition to providing general education about the interventions, there were specific suggestions about how this could be delivered. One participant, for example, indicated that small group education sessions delivered by a non-professional would be favourably received by older adults.

*You have to have a little group session – 3 or 4 people and a person that has knowledge and knows how, they can encourage*. (007)

*In a small group having a person who does all the stuff, but not a professional either, they can encourage—they can say —well, I’ve got the benefits*. (007)

*Long learning curve before you get the hang of it [counter manoeuvres]. Once learnt it is fairly easy*. (010)

Recommendation: Educate older people with OH about how an intervention exerts its affects, or how it might improve their symptoms. Such education could be delivered through group sessions, if feasible.

#### 1.2 Behavioural experiments

This technique focusses on advising how to identify and test hypotheses about the behaviour, its causes and consequences, by collecting and interpreting data e.g. asking patients to perform a specific action to evaluate whether the action had an impact on symptoms.

This technique may also be used to challenge preconceived ideas. Participants appeared to have an established mind-set that either something does not work or that they would be unable to do it. However, by proving that it does work or by demonstrating that the behaviour is not as difficult to perform as expected, people’s motivation to try a therapy may improve.

*If it is shown to be of benefit then I need to pursue that [water bolus]… I’m not convinced that there is a demonstrable benefit to me*. (019)

*It was alright... normally I would think that I can’t drink that amount*. (006)

*I knew I was drinking the water for a purpose... knowing that it was doing me some good*. (003)

*If it [compression garments] is going to help I would suffer it*. (015)

Recommendation: Ask patients to try using an intervention, perhaps while observed in clinic, to demonstrate its feasibility and improve self-efficacy.

### Theme 2—Feedback and monitoring

#### 2.1 Self-monitoring of outcome of behaviour

This strategy requires patients to monitor outcomes which are important to them (e.g. symptoms, daily activity) as part of the strategy to encourage them to modify their behaviour. This may be linked to sub-theme 1.2. It may also be used as a motivator to encourage adherence with the therapy if symptoms are noted to be worsening.

*I thought the glass of water was good, it did make a difference – definitely*. (022)

*I’m not going to NOT do any therapy because I find it burdensome. If it improves my overall life, I’ll do it*. (019)

*If wearing stockings improved symptoms... yes, it would be worthwhile*. (001)

*I’ll do anything if it’s going to help these dizzy spells... if it stops the fainties what more do they want?* (017)

Recommendation: In order to demonstrate effectiveness or tolerability, ask patients to monitor the severity of their symptoms while introducing a therapy to monitor its positive and negative effects.

#### 2.2 Biofeedback

This sub-theme is closely related to sub-theme 2.1, above. However, biofeedback typically refers to objective physiological monitoring, rather than subjective outcomes.

*I’m far more likely to try something positively if I could see that there was a benefit*. (019)

*If it’s [compression stockings] only a tiny benefit I’m not interested*. (010)

*... if it helped blood pressure then I suppose yes I would [wear them]*. (006)

Recommendation: Perhaps more suited to continuous BP monitoring, ask patients to observe their BP while performing physical counter-manoeuvres. Perform a postural BP assessment with and without compression hosiery.

### Theme 3 – Repetition and substitution

#### 3.1 Behavioural practice/rehearsal

Practice of the interventions one or more times in a context or at a time when the performance may not be necessary, may be a valuable strategy to increase habit and skill. An example in this context could be practising physical counter-manoeuvres during clinic visits.

Participants felt that the more they used an intervention, the easier it became to adopt. Then, once a therapy had become part of the older adult’s routine, it became easier to perform.

*Build it up and before you know where you are—it’s part of your routine*. (024)

*Well, I do that now. If I get up, I tense the muscles in my legs before I get up. It is second nature*. (016)

*I think you become accustomed to it because you do it so often. You become more used to putting them [compression stockings] on. The more you do it, the easier it will become*. (001)

Recommendation: Encourage patients to practice an intervention. This could occur in clinic, or, using an individualised approach this could take different forms. E.g. ‘prescribe’ physical counter manoeuvre practice twice per day for a week; practice with nursing staff how to apply and remove compression garments.

#### 3.2 Habit formation

Rehearsal and repetition of a behaviour in the same context repeatedly so that the context elicits the behaviour can be a useful behaviour change strategy. Indeed, embedding a therapy into a daily routine was a common behavioural approach suggested by participants to promote adherence with interventions.

*It’s just a matter of it becoming part of your ritual really*. (024)

*I take water with us when I’m walking now*. (020)

*You can do them [counter-manoeuvres] when you’re washing the dishes*. (006)

*I find it useful to do that [counter-manoeuvres] just standing still*. *If, for example I’m in church standing up*. (008)

*I did do that [counter-manoeuvres] when I was getting up from bed, getting up from sitting watching TV for an hour*. (018)

*I would say three times comfortably [bolus water] – yes, breakfast, midday and the evening*. (010)

*Yes, if I go out, I take a bottle, you know – the little bottles in my bag—a biggish handbag*. (015)

Recommendation: Advise patients to adopt a therapy during specific situations. E.g. perform physical counter manoeuvres while making a hot drink; take a water-bolus while taking morning medication; use compression garments when visiting the supermarket.

### Theme 4—Goals and planning

#### 4.1 Problem solving

Clinicians can prompt their patients to identify factors influencing their behaviour and generate or select strategies that include overcoming barriers and/or increasing facilitators, e.g. a lack of motivation to exercise could be overcome by including a social aspect to exercise.

Data from this theme principally relates to bolus water drinking. One of the main barriers identified by the participants was a dislike of either the taste or temperature of water. Participants suggested they were more likely to continue using bolus water therapy if they could alter the taste (e.g. juice) or drink hot water.

*“But I prefer liquids in other forms – in tea, coffee, lager...*” (008)

“*If I have water in the morning, I have to have it half and half.*” (001) [hot and cold]

*“I drink water, it’s just I like it with a bit juice in.*” (013)

*“If they had some little tablets that made the water fizz – like lemonade or something... make it more palatable.*” (003)

Recommendation: Ask patients to list the perceived barriers to adopting a therapy and help them to formulate a strategy to overcome the barriers.

## Discussion

Non-pharmacologic therapies are preferred over medications by older people with OH; however, their use is limited due to poor tolerability [3, 17]. This study describes patient-derived strategies to improve their uptake and adherence. Strategies were categorised into four core themes, based on the BCT—shaping knowledge, feedback and monitoring, repetition and substitution and lastly goals and planning.

Instruction on how to perform a behaviour should be part of routine clinical care, however, the way in which this is delivered may improve uptake and adherence. This study emphasises the importance of patient education and its role in promoting the uptake and adherence. The optimum delivery method of this education is unknown and may include verbal and written information or signposting to support groups and charities. The data presented here suggest that informing patients about why they are being asked to adopt a therapy should be a core component of patient education. A specific strategy which arose was small-group educational sessions for people with OH, not only for education but also for encouragement. It was also suggested that this could be delivered by a non-professional.

There was a rather pragmatic attitude amongst many of the participants, who felt that if an intervention worked, they would use it. There are two facets to this. Firstly, presenting proof in the form of accessible research evidence and professional experience. However, there is a real lack of research evidence in this area and the therapies used in clinical practice are based upon low quality evidence, scientific theory and expert opinion [13, 14]. The second facet is providing more personalised evidence that an intervention is effective. Based on the responses in the qualitative interviews, participants suggested that they would adhere to an intervention if it improved their symptoms or quality of life. This would depend on an individual’s motivation to try a therapy and adhere to it for long enough to make an impact. An alternative, and more rapid method would be the use of biofeedback. Biofeedback allows an individual to observe physiological responses to different stimuli or conditions. This is an established practice in several areas of medicine (e.g. inhaler technique) but has also been tested in a very small study in people with OH [15]. This study found that standing BP increased when biofeedback was used alongside counter-manoeuvres. This is a potentially very simple tool which could be used in the clinical setting with a BP monitor, but may be more amenable to continuous BP monitoring which is not universally available.

Generating evidence to challenge preconceived ideas also appears to be a useful strategy to encourage uptake and adherence. This was particularly relevant to individuals who felt they would be unable to drink a large glass of water. In this study, participants were surprised how much easier it was than they expected. This could be employed in the clinic setting to assist in overcoming barriers. An additional common barrier to bolus water drinking is also concern around urinary frequency [17]. There were no specific strategies to address this which arose from the data. However, challenging preconceived ideas and trialling the intervention while monitoring its effects are relevant strategies here. The Health on Tap campaign conducted a small study in residential homes to promote the uptake of water drinking. They found that residents who increased their water intake by between 500 ml and 2 l per day visited the loo between zero and two times more than previously [19]. Therefore, a useful strategy to challenge preconceived ideas may be encourage people with OH to trial water therapy while keeping a micturition diary.

Embedding behaviour changes into everyday routines are a commonplace practice, for example, falls prevention exercises embedded in daily routines or taking medications at mealtimes [16]. The data presented here support the role of this technique in improving adherence with therapies for OH. This was a particularly strong theme for water drinking and counter manoeuvres. For water drinking, as well as routine, the data suggested that preparedness was necessary, for example taking water outdoors. Another example given was to perform counter manoeuvres while washing the dishes. Once practiced and familiar, older people with OH suggest that adherence with these therapies may become ‘second nature’.

The final behaviour change strategy which arose was problem solving. This may take on a more personalised approach, exploring barriers with patients and working together to develop a plan to overcome these. This theme was most pronounced within the data relating to bolus water drinking. A common barrier was the taste or temperature of the water, with simple solutions identified to overcome these. Unfortunately, there is no good quality evidence to clarify whether these strategies would reduce the efficacy of the intervention. Only very small, low quality studies have evaluated the effects of the temperature of water and results have been conflicting [20]. Bolus water drinking is thought to exert its effects by reducing the osmolality in the portal circulation and it is unknown whether the addition of juice, flavourings or sugar could reduce this vasopressor response. However, dehydration is known to exacerbate OH, so improving water intake is likely to be of benefit and as described above, if an intervention becomes embedded into daily routine its adherence improves. It is possible therefore, that modifying the taste or temperature of water could be a route to improving the uptake of bolus water drinking.

These strategies presented here require evaluation within the context of experimental studies. The data presented arose from participants who were all recruited from secondary care within one site. Given that participants were within a research study, they may have different motivators, and therefore different strategies, to people who rarely take part in research. Nevertheless, what will be key, is adopting an individualised approach, using this framework as a starting point.

## Conclusions

This study provides specific, clinically relevant recommendations to improve the uptake and adherence of non-drug therapies in older people. These recommendations are derived from older people with OH and are based on an existing evidence-based framework for behaviour change strategies. The strategies include biofeedback, rehearsal, embedding into daily routine and patient education.
